# Analysis of brain atrophy and local gene expression in genetic frontotemporal dementia

**DOI:** 10.1093/braincomms/fcaa122

**Published:** 2020-08-19

**Authors:** Andre Altmann, David M Cash, Martina Bocchetta, Carolin Heller, Regina Reynolds, Katrina Moore, Rhian S Convery, David L Thomas, John C van Swieten, Fermin Moreno, Raquel Sanchez-Valle, Barbara Borroni, Robert Laforce, Mario Masellis, Maria Carmela Tartaglia, Caroline Graff, Daniela Galimberti, James B Rowe, Elizabeth Finger, Matthis Synofzik, Rik Vandenberghe, Alexandre de Mendonça, Fabrizio Tagliavini, Isabel Santana, Simon Ducharme, Chris R Butler, Alex Gerhard, Johannes Levin, Adrian Danek, Giovanni Frisoni, Roberta Ghidoni, Sandro Sorbi, Markus Otto, Mina Ryten, Jonathan D Rohrer, the Genetic FTD Initiative, GENFI, Caroline Greaves, Georgia Peakman, Rachelle Shafei, Emily Todd, Martin N Rossor, Jason D Warren, Nick C Fox, Henrik Zetterberg, Rita Guerreiro, Jose Bras, Jennifer Nicholas, Simon Mead, Lize Jiskoot, Lieke Meeter, Jessica Panman, Janne M Papma, Rick van Minkelen, Yolanda Pijnenburg, Myriam Barandiaran, Begoa Indakoetxea, Alazne Gabilondo, Mikel Tainta, Maria de Arriba, Ana Gorostidi, Miren Zulaica, Jorge Villanua, Zigor Diaz, Sergi Borrego-Ecija, Jaume Olives, Albert Lladó, Mircea Balasa, Anna Antonell, Nuria Bargallo, Enrico Premi, Maura Cosseddu, Stefano Gazzina, Alessandro Padovani, Roberto Gasparotti, Silvana Archetti, Sandra Black, Sara Mitchell, Ekaterina Rogaeva, Morris Freedman, Ron Keren, David Tang-Wai, Linn Öijerstedt, Christin Andersson, Vesna Jelic, Hakan Thonberg, Andrea Arighi, Chiara Fenoglio, Elio Scarpini, Giorgio Fumagalli, Thomas Cope, Carolyn Timberlake, Timothy Rittman, Christen Shoesmith, Robart Bartha, Rosa Rademakers, Carlo Wilke, Hans-Otto Karnarth, Benjamin Bender, Rose Bruffaerts, Philip Van Damme, Mathieu Vandenbulcke, Catarina B Ferreira, Gabriel Miltenberger, Carolina Maruta, Ana Verdelho, Sónia Afonso, Ricardo Taipa, Paola Caroppo, Giuseppe Di Fede, Giorgio Giaccone, Sara Prioni, Veronica Redaelli, Giacomina Rossi, Pietro Tiraboschi, Diana Duro, Maria Rosario Almeida, Miguel Castelo-Branco, Maria João Leitão, Miguel Tabuas-Pereira, Beatriz Santiago, Serge Gauthier, Pedro Rosa-Neto, Michele Veldsman, Paul Thompson, Tobias Langheinrich, Catharina Prix, Tobias Hoegen, Elisabeth Wlasich, Sandra Loosli, Sonja Schonecker, Elisa Semler, Sarah Anderl-Straub, Luisa Benussi, Giuliano Binetti, Michela Pievani, Gemma Lombardi, Benedetta Nacmias, Camilla Ferrari, Valentina Bessi, Cristina Polito

**Keywords:** frontotemporal dementia, atrophy, gene expression, astrocytes, imaging genetics

## Abstract

Frontotemporal dementia is a heterogeneous neurodegenerative disorder characterized by neuronal loss in the frontal and temporal lobes. Despite progress in understanding which genes are associated with the aetiology of frontotemporal dementia, the biological basis of how mutations in these genes lead to cell loss in specific cortical regions remains unclear. In this work, we combined gene expression data for 16 772 genes from the Allen Institute for Brain Science atlas with brain maps of grey matter atrophy in symptomatic *C9orf72*, *GRN* and *MAPT* mutation carriers obtained from the Genetic Frontotemporal dementia Initiative study. No significant association was seen between *C9orf72*, *GRN* and *MAPT* expression and the atrophy patterns in the respective genetic groups. After adjusting for spatial autocorrelation, between 1000 and 5000 genes showed a negative or positive association with the atrophy pattern within each individual genetic group, with the most significantly associated genes being *TREM2*, *SSBP3* and *GPR158* (negative association in *C9Orf72*, *GRN* and *MAPT* respectively) and *RELN*, *MXRA8* and *LPA* (positive association in *C9Orf72*, *GRN* and *MAPT* respectively). An overrepresentation analysis identified a negative association with genes involved in mitochondrial function, and a positive association with genes involved in vascular and glial cell function in each of the genetic groups. A set of 423 and 700 genes showed significant positive and negative association, respectively, with atrophy patterns in all three maps. The gene set with increased expression in spared cortical regions was enriched for neuronal and microglial genes, while the gene set with increased expression in atrophied regions was enriched for astrocyte and endothelial cell genes. Our analysis suggests that these cell types may play a more active role in the onset of neurodegeneration in frontotemporal dementia than previously assumed, and in the case of the positively associated cell marker genes, potentially through emergence of neurotoxic astrocytes and alteration in the blood–brain barrier, respectively.

## Teaser

Altmann *et al.* investigated the concordance between spatial cortical gene expression in healthy subjects and atrophy patterns in genetic frontotemporal dementia. They found that elevated gene expression of endothelial cell and astrocyte-related genes in regions with atrophy, suggesting a role of these cell types in the aetiology of frontotemporal dementia.

## Graphical Abstract

**Graphical Abstract:**
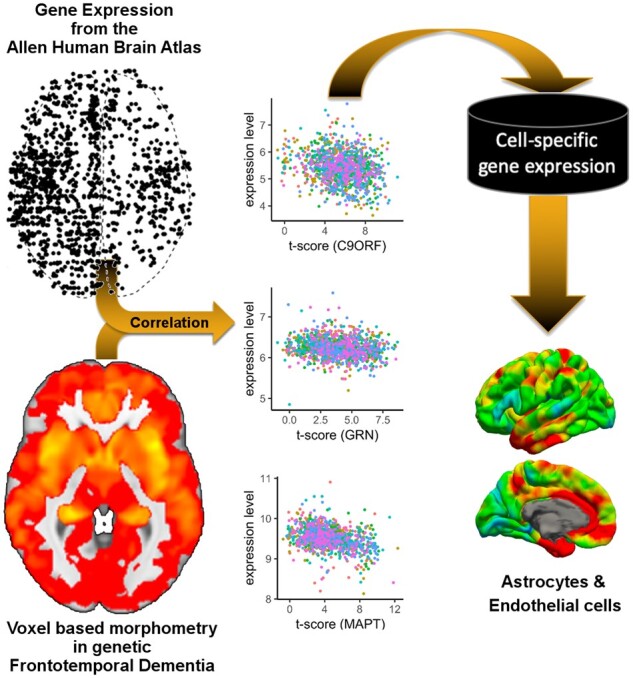

## Introduction

Frontotemporal dementia (FTD) is a heterogeneous neurodegenerative disorder characterized by neuronal loss in the frontal and temporal lobes, with clinical symptoms including behavioural, language and motor deficits (Seelaar *et al.*, 2011). Around 30% of FTD is familial, most commonly caused by autosomal dominant genetic mutations in one of three genes: progranulin (*GRN*), microtubule-associated protein tau (*MAPT*) or chromosome 9 open reading frame 72 (*C9orf72*) (Rohrer *et al.*, 2009). Despite progress in understanding the pathophysiological basis of genetic FTD, the biological basis of how mutations in these genes leads to cell loss in specific cortical regions and subsequently to specific clinical phenotypes is unclear.

An alternative approach to elucidating the molecular biology of autosomal dominant FTD is to study the gene expression profiles of brain regions which are atrophic in symptomatic mutation carriers. This approach has been enabled by publicly available data from the Allen Institute for Brain Science which features post-mortem high-resolution brain-wide gene expression data (i.e. the Allen Atlas) from cognitively normal individuals (Hawrylycz *et al.*, 2012, 2015). In recent years, the Allen Atlas has been successfully integrated with brain maps obtained from case–control studies. For instance, in the case of neurodegenerative disorders, one study investigated the link between gene expression and both regional patterns of atrophy and amyloid deposition, finding a positive correlation of *APP* gene expression and amyloid (Grothe *et al.*, 2018), whilst another study showed that expression of the *MAPT* gene was associated with changes in functional connectivity in Parkinson’s disease (Rittman *et al.*, 2016).

In this work, we combine gene expression data from the Allen Atlas with brain maps of grey matter atrophy in symptomatic *C9orf72*, *GRN* and *MAPT* mutation carriers from the genetic FTD initiative (GENFI) study compared with non-carriers (Cash *et al.*, 2018). The aim of this study was to investigate the molecular basis of the atrophy pattern in mutation carriers. We firstly investigated the spatial overlap between grey matter atrophy in each of the three genetic groups and the gene expression of the corresponding gene. We then aimed to identify which genes showed a high spatial correspondence between their expression throughout the brain and the atrophy pattern in each genetic group. We hypothesized that these genes or groups of genes may implicate molecular processes or brain cell types that explain why these regions are particularly vulnerable in FTD.

## Materials and methods

### Allen Human Brain Atlas data

We used the brain-wide microarray gene expression data generated by the Allen Institute for Brain Science (downloaded from http://human.brain-map.org/) (Hawrylycz *et al.*, 2012, 2015). The dataset consists of a total of 3702 microarray samples from six donors (one female). Each sample comprised 58 692 gene probes and provides coordinates in MNI152 space. The gene expression data have been normalized and corrected for batch effects by the Allen Institute (TECHNICAL WHITE PAPER: MICROARRAY DATA NORMALIZATION, 2013). We first restricted the set of samples to the left hemisphere (six in total) and to cortical regions based on the provided slab type (‘*cortex*’), retaining only samples with a maximal distance of 3 mm to a cortical region of interest obtained by a parcellation (Cardoso *et al.*, 2015) of the study template used in Cash et *al.* (2018). After additionally removing samples being annotated by the Allen Institute as non-cortical samples (e.g. CA1 field), 1248 microarray samples were available in total. Next, as previously described (Richiardi *et al.*, 2015) we reannotated all microarray probe sequences with gene names using Re-Annotator (Arloth *et al.*, 2015). We excluded probes that sampled more than one gene (*N* = 1512), were mapped to intergenic regions (*N* = 5013) or could not be mapped to any genomic region (*N* = 1569), leaving 50 598 probes covering 19 980 unique genes. Furthermore, we removed probes that were marked as expressed in <300 of the 1248 cortical samples (*N* = 13 941). Thus, the analysis was carried out using 36 657 microarray probes covering 16 772 distinct genes.

### Image data preparation

**Figure 1:**
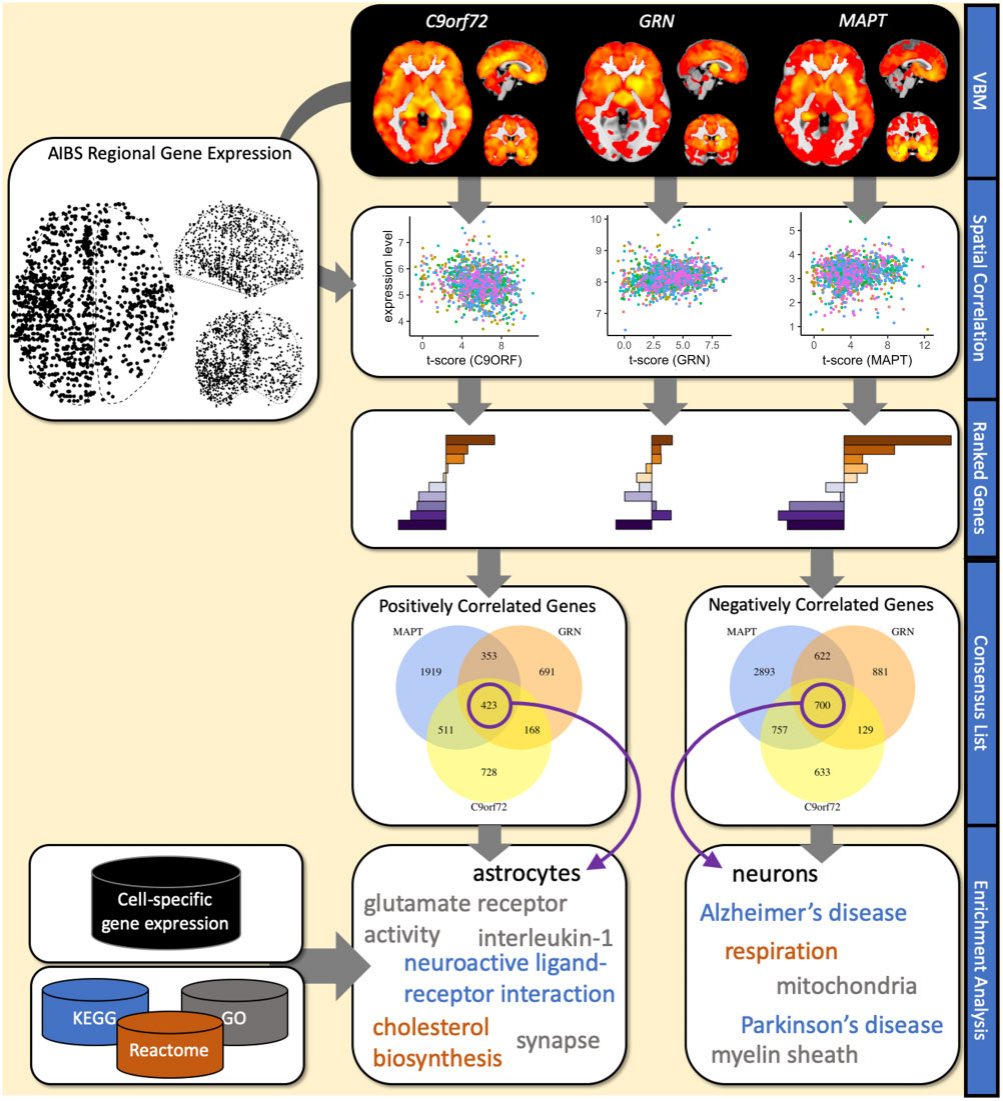
**Analysis overview.** Starting point of the analysis are the statistical maps from VBM analyses comparing healthy controls and symptomatic FTD mutation carriers in *C9orf72*, *GRN* and *MAPT* from Cash *et al.* (2018). For each statistical map the spatial association with the expression levels 16 772 genes (represented by 36 657 gene expression probes) is computed using data from the Allen Institute for Brain Science gene expression atlas. The resulting gene ranking provides lists of genes that are either significantly (P_FDR_<0.05) positively or negatively correlated with the atrophy pattern. Two consensus lists were generated from the three lists of positively correlated and negatively correlated genes, respectively. Resulting gene lists were analysed for enrichment of signature genes for brain cell types, such as neurons, microglia or astrocytes and biological pathways.

To quantify the amount of atrophy in carriers of FTD mutations, we used results from a voxel-based morphometry (VBM) analysis of the GENFI dataset (Cash *et al.*, 2018). In particular, in this analysis we used the maps showing the voxel-wise *t*-statistic (*t*-maps) comparing symptomatic mutation carriers (*MAPT*: *N* = 10; *GRN*: *N* = 12; *C9orf72*: *N* = 25) to non-carriers (*N* = 144) (Fig. 1; *top*). Here, higher *t*-scores signify higher average atrophy in the symptomatic group analysis. A mean bias corrected image from all the normalized T1 images in the GENFI study served as a study template. This template was warped into Montreal Neurological Institute and Hospital space using the non-rigid registration based on fast free form deformation implemented in NiftyReg (version 10.4.15) (Modat *et al.*, 2010). The obtained transformation was then applied to each of the three *t*-maps. For each Montreal Neurological Institute and Hospital coordinate of the eligible cortical gene expression samples we located the corresponding voxel in the *t*-map and obtained the *t*-value centred on those Montreal Neurological Institute and Hospital coordinates. This procedure was carried out for each of the three *t*-maps and resulted in a 1248 by three matrix, i.e. each gene expression sample was linked to three *t*-scores from the VBM analysis (one for each FTD gene).

### Association analysis

The overall analysis is depicted in Fig. 1. We analysed the association between atrophy and gene expression in a quasi-non-parametric fashion. For a given atrophy map and a given probe, we approximated the Spearman (or rank) correlation (*ρ*) between the local *t*-score and the gene expression level separately for each of the six donors while accounting for spatial autocorrelation. This was achieved by first converting *t*-scores and expression levels into ranks, respectively, and using linear regression to approximate the Spearman rank correlation (Conover and Iman, 1981); and second by adjusting this linear model using spatial eigenvectors that account for the spatial relationship between samples. More precisely, we used the method of spatial eigenvector mapping [reviewed in Dormann *et al.* (2007)] to adjust for spatial autocorrelation. In this approach, one first checks the residuals of the linear model for presence of spatial autocorrelation using Moran’s I (Moran, 1950), which assumes spatial stationarity of the autocorrelation across the brain. If there was evidence for spatial autocorrelation (as indicated by *P* < 0.05 obtained with the moran.test function in the spdep R package), then the first spatial eigenvector was added as a confound variable to the linear model. Next, if the residuals of the resulting extended linear model continued to show evidence for spatial autocorrelation, then further eigenvectors were added until the residual was free from evidence of spatial autocorrelation (i.e. *P* > 0.05). To accelerate the process, spatial eigenvectors were added to the model in batches of 5 and up to 150 spatial eigenvectors were considered. Spatial eigenvectors for this analysis were obtained from the 10-nearest neighbour graph constructed using the geodesic distances between gene expression samples, which were generated for each donor brain. From the resulting linear model, we extracted the *t*-scores and the corresponding *P*-value for the association between atrophy and gene expression. Next, we combined the six *P*-values into a single meta *P*-value using the weighted sum of *Z*-scores (Stouffer’s) method. On purpose, we restricted weights to −1 and +1 to only indicate the direction of association, and to avoid over-emphasizing the impact of donors with more gene expression samples. This procedure was carried out for each for the 36 657 probes and each of the three genetic group atrophy maps. *P*-values in each of the three resulting lists were corrected for multiple testing using the method by Benjamini and Hochberg (1995) for false discovery rate (FDR).

Significantly positively correlated genes (i.e. higher gene expression is linked to higher atrophy) were those where any probe targeting the gene reached an FDR-corrected *P*-value < 0.05 and a positive *Z*-score; likewise significantly negatively correlated genes (i.e. higher gene expression is linked to lower atrophy) were required to have an FDR-corrected *P*-value < 0.05 and a negative *Z*-score for any of the probes targeting that gene. We also created two overlap lists, one containing the overlap of genes in the three positive lists, the other the overlap of genes in all three negative lists. In the following we refer to these two lists as *consensus* lists.

### Overrepresentation analysis

To identify cellular pathways or cellular processes and cell-type signature genes that may be enriched in the significant gene lists, we conducted an overrepresentation analysis. We obtained the following gene sets from the MSigDB database version 6.1 (date accessed 23 November 2017): (i) Gene Ontology (GO) (*N* = 5917 sets), (ii) REACTOME (*N* = 674 pathways) and (iii) KEGG (*N* = 186 pathways). We used Fisher’s exact test to compute the odds ratio (OR) and *P*-value for overrepresentation of genes in a given set. All tests were carried out using the 16 772 cortex expressed genes as the background set. For each of the three gene lists (i.e. one per FTD gene) and the two consensus lists we corrected the *P*-values using the FDR correction based on Benjamini and Hochberg across all 6777 gene sets.

To determine whether the expressed genes implicate a specific class of brain cell types, in addition to enrichment analysis for GO terms and pathways, we conducted an enrichment analysis for marker gene lists for six brain cell-types based on RNA sequencing of purified human cells (Zhang *et al.*, 2016). In brief, we generated cell-type specific lists from the available average expression levels per cell-type: only genes with expression levels exceeding 2.5 fragments per kilobase were included and genes were required to show an enrichment of at least 3.0 (i.e. fragments per kilobase in target cell-type divided by average fragments per kilobase in non-target cell-types).

### Expression-weighted cell-type enrichment analysis

In an additional analysis we sought to identify potential brain cell types that were implicated by all three FTD genes. To this end we conducted expression-weighted cell-type enrichment (EWCE) analysis (Skene and Grant, 2016) on the two consensus lists using a recently published dataset of brain single-cell sequencing data in the mouse brain that identified 265 different cell types (www.mousebrain.org) (Zeisel *et al.*, 2018). From this dataset we removed 76 cell types that were not directly brain related (e.g. cell belonging to enteric nervous system or the spinal cord), leaving 189 different cell-type signatures. Each of the cell types is also attributed with a high-level annotation (astrocytes, ependymal, immune, neurons, oligos, vascular). In brief, from the single cell mouse dataset we used only genes that had a unique human homolog (1-to-1 mapping). Then, we analysed the two consensus lists separately for high-level cell-type enrichment using EWCE with correction for gene length and GC content. EWCE was executed twice, once using high-level annotations and once using the 189 cell-type annotations. *P*-values are based on 100 000 permutations and enrichment *P*-values were corrected for multiple testing using the Benjamini and Hochberg method for FDR. We used the available R package for EWCE.

### Interpretation of association, overrepresentation and EWCE analyses

Overall, the type of gene expression profiling used in Allen Atlas is based on measuring bulk expression of tissue samples, i.e. a group of diverse cell types is sampled at once and the resulting expression profile represents the group average of this set of cells and their states. Thus, in these analyses, genes positively correlated with atrophy indicate potential cellular processes and cell types that promote atrophy in genetic FTD. Conversely, genes that are negatively correlated with atrophy indicate potential cellular processes and cell types that confer resilience to disease-related neurodegeneration.

### Data availability

Statistical maps of the VBM analysis were obtained from Cash *et al.* (2018) and are available upon request from the lead author of that study. Cortical gene expression data were obtained from the Allen Institute of Brain Sciences and can be obtained at http://human.brain-map.org/. Code for pre-processing expression data and conducting the regional association analysis with adjustment for spatial autocorrection can be accessed through: https://github.com/andrealtmann/AIBS_FTD.

## Results

We tested 16 776 genes (from 36 657 microarray probes) for their association with atrophy pattern across the cortex in genetic FTD (Fig. 1). The numbers of significant probes (P_FDR_<0.05) and genes with their direction for each of the three FTD genes are listed in Table 1. The association results per probe are available as Supplementary material (Dataset 1). The number of included spatial eigenvectors to adjust for spatial autocorrelation ranged from one to 25, with a median (inter quartile range) of 5 (1–10).

**Table 1:** Numbers of significant probes and genes and their direction of association after Benjamini and Hochberg correction for FDR

	Probes		Genes	
	Positive	Negative	Positive	Negative
*C9orf72*	2208	2698	1830	2219
*GRN*	2078	2849	1635	2332
*MAPT*	4527	7530	3206	4972
Overlap	539	784	423	700

### C9orf72

**Figure 2:**
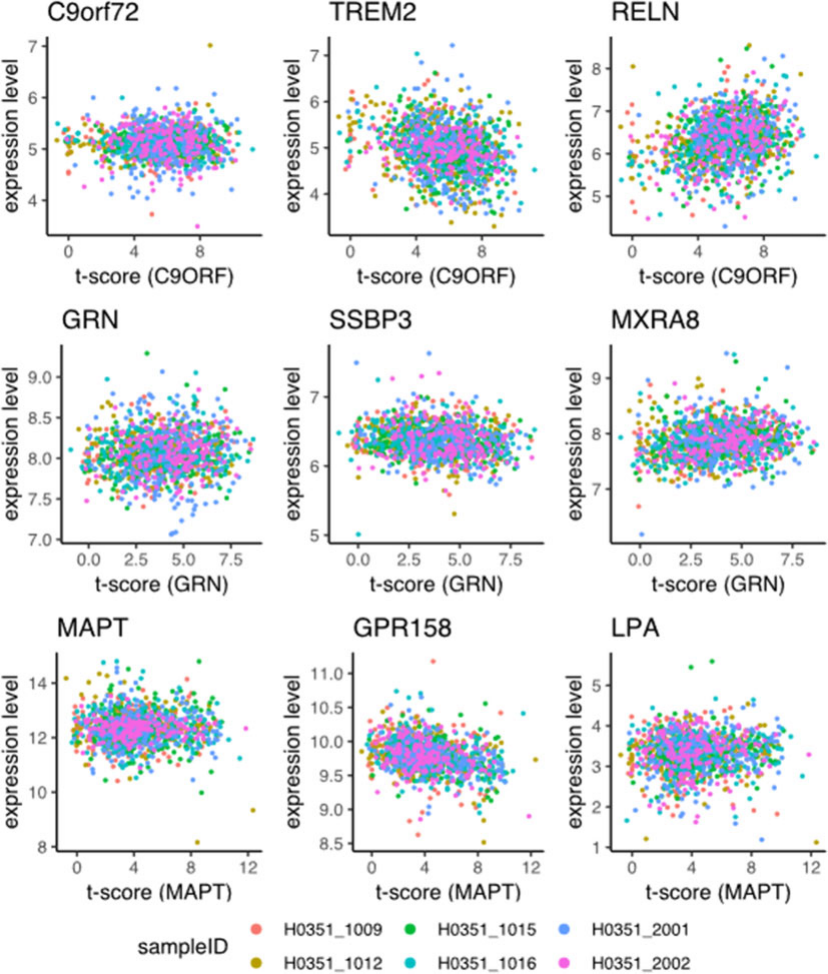
**Selected scatterplots between *t*-scores and gene expression levels.** Rows correspond to the three different FTD genes (*C9orf72*, *GRN* and *MAPT*). *x*-Axis shows to the *t*-value in the corresponding statistical VBM maps (Fig. 1) from Cash *et al.* (2018) at positions where microarray samples were obtained in the Allen Atlas; the *y*-axis represents the expression levels in the Allen Atlas after adjustment for spatial autocorrelation for the gene named at the top of the scatter plot. Each point represents one microarray sample and the colour indicates the donor ID. In the first column expression of the corresponding FTD genes is studied: *C9orf72* (CUST_7641_PI416261804), *GRN* (CUST_13046_PI416261804) and *MAPT* (A_24_P224488). The second column shows the most strongly negatively correlated genes, i.e. genes with high expression in brain regions showing little atrophy: *TREM2* (A_23_P167941), *SSBP3* (A_23_P500333) and *GPR158* (A_24_P349117). The third column shows the genes where expression level and atrophy positively correlate the strongest: *RELN* (A_24_P309095), *MXRA8* (A_23_P32444) and *LPA* (A_23_P95221).

The strongest association between *C9orf72* expression and the atrophy pattern in symptomatic chromosome 9 open reading frame 72 repeat extension carriers was measured with microarray probe CUST_7641_PI416261804, which showed a non-significant negative association *z* = −0.27 (P_FDR_=0.23; Fig. 2).

The most negatively associated gene was *TREM2* (triggering receptor expressed on myeloid cells 2; represented by probe A_23_P167941; *z* = −6.26; P_FDR_=6.94e−07; Fig. 2; Table 2; Supplementary Dataset 1). Top-ranked gene sets based on the significantly negatively correlated genes include genes related to mitochondria (GO_MITOCHONDRIAL_PART; OR = 3.00; P_FDR_=1.77e−35) and the respiratory chain (GO_RESPIRATORY_CHAIN; OR = 9.92; P_FDR_=5.91e−19; Supplementary Dataset 2). Notably, KEGG pathways for neurodegenerative disorders were highly enriched (KEGG_PARKINSONS_DISEASE OR = 7.35 P_FDR_=8.74e−20; KEGG_HUNTINGTONS_DISEASE OR = 4.99 P_FDR_=8.18e−18; KEGG_ALZHEIMERS_DISEASE OR = 4.15 P_FDR_=4.38e−14). Among brain cell types, there was a strong enrichment for neuronal genes (OR = 1.7; P_FDR_=1.13e−12) and microglia (OR = 1.59; P_FDR_=2.53e−06; Supplementary Dataset 3).

**Table 2:** Top 10 most positively and negatively correlated genes with atrophy patterns in genetic FTD

Rank	*C9orf72*		*GRN*		*MAPT*	
	Negative	Positive	Negative	Positive	Negative	Positive
1	*TREM2*	*RELN*	*SSBP3*	*MXRA8*	*GPR158*	*LPA*
2	*RAB11FIP5*	*SCAPER*	*KIF5A*	*DCHS2*	*ITGBL1*	*NUPR1*
3	*FPR1*	*MXRA8*	*HLA-DPA1*	*NDNF*	*STRBP*	*STOM*
4	*EPDR1*	*MFAP4*	*PGM5P2*	*CRIP1*	*KCTD9*	*DCAF15*
5	*FXR1*	*FMOD*	*WIBG*	*FBLN2*	*ERRFI1*	*ARHGAP36*
6	*KATNAL2*	*CLIC5*	*MGC39584*	*CNR1*	*F7*	*ALCAM*
7	*FAM3B*	*SRGAP1*	*CPLX2*	*TGFBI*	*CD99L2*	*PON3*
8	*PLA2G7*	*NDNF*	*POU6F2*	*RELN*	*GLRX*	*C1orf228*
9	*ABRACL*	*DCLK3*	*C2*	*PODXL*	*ST8SIA5*	*MXRA8*
10	*HIST1H4H*	*ARHGAP36*	*NRG1*	*ARHGAP36*	*EMB*	*DRAXIN*

Rows correspond to the three genes and an overlap between all three lists.

The most significantly positively correlated gene was reelin; represented by probe A_24_P309095; *z* = 7.94; P_FDR_=1.92e−11; Fig. 2, Table 2. Top-ranked GO terms for positively correlated genes included vascular development and glial cell differentiation (GO_REGULATION_OF_VASCULATURE_DEVELOPMENT OR = 2.28 P_FDR_=9.95e−04; GO_GLIAL_CELL_DIFFERENTIATION OR = 2.66 P_FDR_=1.13e−03; Supplementary Dataset 2). Among brain cell types, there was a strong enrichment for genes associated with oligodendrocytes (OR = 9.37; P_FDR_=3.1e−54), endothelial cells (OR = 2.4; P_FDR_=3.63e−10) and mature astrocytes (OR = 1.62; P_FDR_=1.23e−05; Supplementary Dataset 3).

### Progranulin

The strongest association between *GRN* expression and the atrophy pattern in symptomatic GRN mutation carriers was measured with microarray probe CUST_13046_PI416261804, which showed a non-significant positive association *z* = 1.31 (P_FDR_=0.13; Fig. 2).

The most significantly negatively correlated gene was single-stranded DNA-binding protein 3; *z* = −6.73; P_FDR_=1.57e−07; Fig. 2; Table 2. For the negatively correlated genes, significantly enriched gene sets featured the respiratory chain (e.g. REACTOME’s RESPIRATORY_ELECTRON_TRANSPORT OR = 5.04 P_FDR_=6.13e−06; Supplementary Dataset 2). As with chromosome 9 open reading frame 72, the three KEGG pathways for neurodegenerative disorders were enriched (Alzheimer’s OR = 2.38 P_FDR_=3.64e−03; Parkinson’s OR = 3.09 P_FDR_=2.17e−04; Huntington’s OR = 2.26 P_FDR_=5.32e−03). Among brain cell types, there was a strong enrichment for neuronal genes (OR = 1.71; P_FDR_=2.06e−13) and microglia (OR = 1.68; P_FDR_=4.10e−08; Supplementary Dataset 3).

The most significantly positively associated gene was matrix remodelling associated 8; *z* = 6.36; P_FDR_=5.87e−07; Fig. 2; Table 2. Top-ranked GO terms (Supplementary Dataset 2) for positively correlated genes are related to the extracellular matrix (GO_EXTRACELLULAR_MATRIX OR = 3.79 P_FDR_=1.25e−18), vascular development, (GO_VASCULATURE_DEVELOPMENT OR = 2.68 P_FDR_=4.16e−11) and response to wounding (GO_RESPONSE_TO_WOUNDING OR = 2.45 P_FDR_=9.19e−10). Again, genes related to oligodendrocytes showed the strongest enrichment (OR = 9.47; P_FDR_=6.1e−53), followed by mature astrocytes (OR = 3.19; P_FDR_=6.62e−32) and endothelial cells (OR = 4.21; P_FDR_=1.18e−28; Supplementary Dataset 3).

### Microtubule-associated protein tau

The strongest association between *MAPT* expression and the atrophy pattern in symptomatic MAPT mutation carriers was measured with microarray probe A_24_P224488, which showed a non-significant positive association *z *= 0.867 (P_FDR_=0.15; Fig. 2).

The most significantly negatively correlated gene was G-protein-coupled receptor 158; *z* = −9.32; P_FDR_=1.06e−16; Fig. 2; Table 2. As in the case of *C9orf72*, the significantly negatively correlated genes showed enrichment for mitochondria (GO_MITOCHONDRIAL_PART; OR = 2.11; P_FDR_=2.90e−22) and cellular respiration (GO_CELLULAR_RESPIRATION; OR = 3.69; P_FDR_=1.72e−11; Supplementary Dataset 2). Notably, KEGG pathways for neurodegenerative disorders were enriched (Parkinson’s OR = 6.05 P_FDR_=2.75e−16; Alzheimer’s OR = 3.37 P_FDR_=7.76e−11; Huntington’s OR = 3.08 P_FDR_=4.47e−10). Within the cell types, neuronal genes were strongly enriched (OR = 2.26; P_FDR_=2.22e−46).

The most significantly positively associated gene was lipoprotein(a); *z* = 8.32; P_FDR_=6.48e−11; Fig. 2; Table 2. Significantly positively correlated genes are enriched for genes related to nervous system development (GO_REGULATION_OF_NERVOUS_SYSTEM_DEVELOPMENT; OR = 1.83; P_FDR_=3.03e−08) and gliogenesis (GO_GLIOGENESIS; OR = 2.66; P_FDR_=2.57e−06). Furthermore, the positively correlated genes were strongly enriched for genes related to mature astrocytes (OR = 5.33, P_FDR_=5.66e−102) and moderately enriched for oligodendrocytes related genes (OR = 1.78, P_FDR_=2.46e−04; Supplementary Dataset 3).

**Figure 3:**
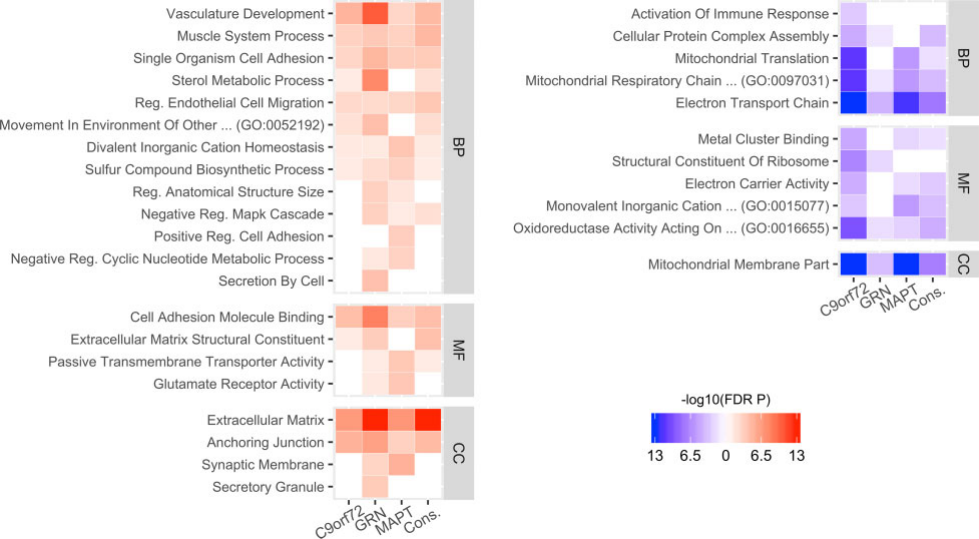
**Summary of GO enrichment analysis.** Selected GO enrichment results for the three genetic groups and the consensus lists (Cons.). The left panel shows enrichment for genes showing a significant positive association with atrophy while the right panel depicts enrichments for negatively correlated genes. Significance of the enrichment is colour-coded: more saturated colours signify smaller *P*-values and non-significant results (i.e. FDR corrected *P*-value > 0.05) appear white. Depicted GO terms were reduced by filtering based on the number of genes (<500) and by clustering terms with similar meaning using the measure of semantic similarity introduced by Wang *et al.* (2007). The full list of results is available in Supplementary Dataset 2. BP: biological process; MF: molecular function; CC: cellular component.

Enrichment analyses for GO terms for each genetic group and the consensus list are summarized in Fig. 3.

### Consensus lists

From the gene lists obtained for each of the FTD gene atrophy maps we created two consensus lists: one comprising 423 genes that were significantly positively correlated with atrophy in all three FTD genes and one list comprising the 700 genes that were significantly negatively correlated in all three maps (Fig. 1). Using these lists, we aimed to identify a common theme underlying the atrophy in the three causative genes.

**Figure 4:**
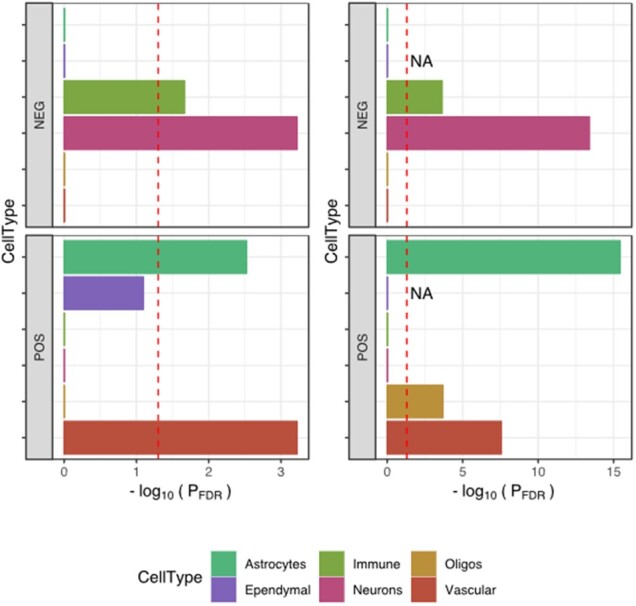
**Cell-type enrichment in consensus gene lists.** Two different methods were used to investigate cell-type signatures in the two consensus lists (POS: gene expression is positively correlated with atrophy; NEG: gene expression is negatively correlated with atrophy). The left panels used the EWCE method together with single-cell RNA sequencing data from mouse brains (Zeisel *et al.*, 2018); the enrichment is summarized as −log_10_(P_FDR_). The right panels used a classic over-representation analysis based on Fisher’s exact test with cell-type signature genes obtained from bulk RNA sequencing of purified human cells (Zhang *et al.*, 2016). The red dashed lines indicate statistical significance after FDR correction [i.e. −log_10_(0.05)]. Both approaches demonstrated a strong neuronal and immune signature in negatively correlated genes (upper panels) and a strong vascular and astrocyte signature in positively correlated genes (lower panels). Cell types are presented in alphabetical order from top to bottom.

The high-level analysis EWCE showed enrichment for vascular marker genes (Fig. 4; *Z*-score = 9.44; P_FDR_=5.99e−05), astrocyte genes (Fig. 4; *Z*-score = 3.24; P_FDR_=0.0033) among genes with positive association to atrophy severity. Genes negatively correlated with atrophy were highly enriched for neuronal genes (*Z*-score = 6.65; P_FDR_<5.99e−05) and immune cell genes (*Z*-score = 2.56; P_FDR_=0.019). The detailed EWCE analysis of the 189 cell-types confirmed the high-level analysis: nearly all cell-types belonging to the astrocyte and vascular classes showed significant enrichment in the positive consensus list (Supplementary Dataset 4). The negative consensus list was enriched for microglia and for inhibitory as well as excitatory neurons.

These results were further confirmed using cell-type marker genes derived from RNA sequencing of purified human cells (Zhang *et al.*, 2016) (Fig. 4; right column), with the strongest enrichment being seen for astrocyte marker genes (OR = 3.89; P_FDR_=3.7e−16). In addition, oligodendrocytes marker genes were enriched among genes with positive association to atrophy severity (OR = 2.61; P_FDR_=2.04e−04).

The remaining gene set analyses confirmed results obtained with the individual gene lists (Fig. 3; Supplementary Dataset 2).

## Discussion

We investigated the gene expression correlates of the cortical regions specifically atrophic in the three main genetic causes of FTD. Whilst there was no association with expression of the gene itself (i.e. *C9orf72*, *GRN* and *MAPT*), our analysis revealed that groups of genes commonly associated with astrocytes and endothelial cells showed higher expression levels in regions with more atrophy and genes commonly associated with neurons and microglia showed higher expression levels in the relatively spared regions.

Astrocytes are the most abundant cell-type in the human CNS and carry out a plethora of functions including biochemical support for the blood–brain barrier (BBB)-forming endothelial cells, trophic support for neurons, regulation of extracellular ion balance and participation in repair processes of the brain following injuries. Astrocytes reacting to injuries in the central nervous system, reactive astrocytes, are characterized by expression of glial fibrillary acidic protein (GFAP). Depending on the context, such reactive GFAP-positive astrocytes can be neurotoxic (Liddelow and Barres, 2015; Liddelow *et al.*, 2017) or neuroprotective (Anderson *et al.*, 2016). There are already multiple lines of evidence linking astrocyte (dys-)function to neurodegeneration (Rodríguez *et al.*, 2009; Phatnani and Maniatis, 2015; Sofroniew, 2015) and to FTD in particular. For instance, histopathological studies in FTD have shown that severity of astrocytosis and astrocytic apoptosis correlates with the degree of neuronal loss as well as with the stage of the disease (Broe *et al.*, 2004). In addition, astrocyte reactivity appears to be region specific in that higher numbers of reactive (GFAP-positive) astrocytes were found in the frontal and temporal cortices of FTD patients compared to controls (Martinac *et al.*, 2001). These observations extend to the CSF where levels of GFAP were increased in various neurodegenerative disorders compared to cognitively normal adults with the highest levels in FTD patients (Ishiki *et al.*, 2016). Martinac et *al.* (2001) found that degrading astrocytes were inversely correlated with cerebral blood flow in FTD. However, more importantly, astrocytes derived from induced pluripotent stem cells of patients with mutations in *MAPT* were found to demonstrate increased vulnerability to oxidative stress and exhibit disease-associated gene-expression changes (Hallmann *et al.*, 2017). Co-culture experiments of such modified FTD astrocytes with previously healthy neurons led, among other things, to increased oxidative stress in these neurons. Hence, taken together, astrocyte reactivity and activation of GFAP may well co-occur with disease onset and progression in FTD.

A second cell-type for which we observed consistent enrichment in brain regions with atrophy were endothelial (or vascular) cells, whilst the overrepresentation analysis was also enriched for vascular terms (circulatory system development). Furthermore, matrix remodelling associated 8 was found in the top 10 for all three genetic group lists—this gene codes for limitrin, a protein specific to the glia limitans, a component of the BBB. Overall, this suggests a role of the BBB in regional vulnerability in genetic FTD. Dysfunction of the BBB has been implicated in the aetiology of many neurodegenerative disorders (Sweeney *et al.*, 2018), with BBB permeability previously found to be abnormal in patients with FTD (Janelidze *et al.*, 2017). The BBB is ubiquitous in the cortex, however, our results based on bulk tissue gene expression profiling would suggest regional differences in density of endothelial cells and their link to disease specific atrophy patterns. Indeed, vascular structure in the brain is known to be heterogenous and characterized by differential pathophysiological responses (Cavaglia *et al.*, 2001).

Brain regions that were relatively spared in FTD showed higher expression of neuron-related genes. The detailed EWCE analyses showed that both inhibitory and excitatory neuron types were enriched (Supplementary Dataset 4). Higher expression in spared regions implies lower gene expression in affected regions, which by itself could be a source for increased vulnerability since minor perturbations in gene expression may have a disproportionate effect. However, given that gene expression data was generated using bulk tissue sampling and that neurons have a significant spatial spread via extended dendritic and axonal processes, the observed enrichment signal could originate from differences between mRNA located in the soma and in dendrites, respectively. A recent pathway analysis of 2028 differentially dendritically localized mRNA isoforms showed strong enrichment for GO terms such as respiratory electron transport chain (Middleton *et al.*, 2019), similar to our enrichment pattern. Indeed, a *post hoc* enrichment analysis of these dendritically localized mRNA isoforms [Supplementary Table 2 from Middleton *et al.* (2019)] confirmed strong enrichment in all three individual gene lists (OR > 1.5, *P* < 1.07e−10) as well as the consensus list (OR = 1.92, *P* = 5.35e−09). Thus, this result may suggest increased and decreased dendrite density in spared and affected regions, respectively. Neuron morphology is very diverse and notably neurons with selective vulnerability in FTD such as von Economo neurons and fork cells (Kim *et al.*, 2012; Seeley *et al.*, 2012) are morphologically characterized by large cell bodies and limited branching of dendrites.

In addition to neuron-related genes, there was also an enrichment of immune system-related genes (e.g. microglia genes) in brain regions with reduced atrophy. Recent works using ^11^C-PK-11195 PET, a marker of activated microglia, have linked regional patterns of neuroinflammation to protein aggregation in FTD (Bevan-Jones *et al.*, 2020; Malpetti *et al.*, 2020). Furthermore, a number of genes associated with immune function were strongly negatively correlated with gene expression in the association analysis. In particular, *TREM2* showed the strongest negative association in the *C9orf72* list and was also significantly associated in the *GRN* and *MAPT* lists (*z* < −4.39, P_FDR_<2.68e−04); this is a transmembrane receptor that participates in modulation of the immune response. Heterozygous variants in *TREM2* are known to be a risk factor for Alzheimer’s disease (Guerreiro *et al.*, 2013) and levels of the TREM2 protein have been shown to be abnormal in FTD (Heywood *et al.*, 2018; Woollacott *et al.*, 2018).

Molecular pathways related to mitochondria were also negatively correlated with gene expression in the analysis. Mitochondrial abnormalities have recently been associated with FTD (Lau *et al.*, 2018), particularly with *C9orf72* mutations (Lopez-Gonzalez *et al.*, 2016; Choi *et al.*, 2019), although little is known at present about the exact molecular mechanisms underlying such dysfunction.

The three individual genetic group lists as well as the consensus list feature significant enrichment for KEGG pathways for Alzheimer’s, Parkinson’s and Huntington’s disease. This may be due to a strong overlap in dendritically localized mRNA isoforms and these pathways. Notably, the KEGG pathway for ALS, which features only 53 genes, did not reach statistical significance after FDR correction in any of the three groups (OR = 2.12, *P* = 0.0198, P_FDR_=0.59 in the *GRN* list, *P* > 0.05 in the *MAPT* and *C9orf72* lists).

Summarizing, we detected higher levels of astrocyte and endothelial cell-related genes in regions with neurodegeneration in FTD and we found that genes associated with neurons and microglia were more enriched in brain regions that are spared in FTD. Furthermore, there was enrichment for genes that are associated with mitochondria, particularly cellular respiration, in regions that are not affected by atrophy. These results confirm earlier results from an unbiased proteomic screen of tissue samples where the modules related to synapse (M1), mitochondrion (M3) and neuron differentiation (M8) showed negative associations with clinicopathological traits in FTD, i.e. these three modules were consistently negatively correlated with FTD pathology (Umoh *et al.*, 2018). Consistent with our results, there was a positive association between clinicopathological traits and modules M5 (Extracellular matrix) and M6 (Response to biotic stimulus), which both showed strong cell-type enrichment for astrocyte-related proteins. Notably, gene sets associated with the extracellular matrix were also enriched in our analysis with regions showing increased atrophy in FTD (Supplementary Dataset 2). While Umoh *et al.* (2018) interpreted the negative (and positive) correlations in part with a disease-related shift in cell population in the sampled region of interests, our results extend this observation to regional cell-type densities since the gene expression samples were obtained from six cognitively normal subjects.

In summary, our analysis of bulk tissue expression data suggests that cortical regions exhibiting the most severe atrophy in genetic FTD may be those with higher expression of astrocyte and endothelial cell-related genes in healthy subjects. Regional cell-density measurements obtained from post-mortem histological samples of healthy subjects would be required to confirm this finding. However, our observation fits well with increased BBB permeability in FTD patients and recent findings of the neurotoxic potential of astrocytes (Liddelow *et al.*, 2017). We hypothesize that the distinct regional atrophy pattern in genetic FTD may be driven by regions with naturally increased astrocyte density where these universal astrocyte neurotoxic effects come to bear with higher frequency. Thus, neurodegeneration may be the result of age-related increase in neurotoxic (A1) and senescent astrocytes, which lost many normal astrocytic functions.

## Funding

A.A. holds a Medical Research Council eMedLab Medical Bioinformatics Career Development Fellowship. This work was supported by the Medical Research Council (grant number MR/L016311/1). The Dementia Research Centre is supported by Alzheimer's Research UK, Brain Research Trust and The Wolfson Foundation. This work was supported by the National Institute for Health Research Queen Square Dementia Biomedical Research Unit, the National Institute for Health Research UCL/H Biomedical Research Centre and the Leonard Wolfson Experimental Neurology Centre (LWENC) Clinical Research Facility as well as an Alzheimer's Society grant (AS-PG-16-007). J.D.R. is supported by a Medical Research Council Clinician Scientist Fellowship (MR/M008525/1) and has received funding from the National Institute for Health Research Rare Disease Translational Research Collaboration (BRC149/NS/MH). This work was also supported by the Medical Research Council UK GENFI grant (MR/M023664/1) and the Bluefield Project. M.R. holds a Medical Research Council Clinician Scientist Fellowship (grant number MR/N008324/1). R.H.R. was supported through the award of a Leonard Wolfson Doctoral Training Fellowship in Neurodegeneration. This work was supported by Italian Ministry of Health (CoEN015 and Ricerca Corrente). Several authors of this publication (J.C.v.S., M.S., R.S-V., A.D., M.O., J.B.R.) are members of the European Reference Network for Rare Neurological Diseases—Project ID No. 739510. This project was supported, in part, via the European Union’s Horizon 2020 research and innovation programme grant 779257 “Solve-RD” (to M.S.).

## Supplementary material

Supplementary material is available at *Brain Communications* online.

## Competing interests

The authors report no competing interests.

## Appendix

List of GENFI consortium authors: Caroline Greaves, Georgia Peakman, Rachelle Shafei, Emily Todd, Martin N. Rossor, Jason D. Warren, Nick C. Fox, Henrik Zetterberg, Rita Guerreiro, Jose Bras, Jennifer Nicholas, Simon Mead, Lize Jiskoot, Lieke Meeter, Jessica Panman, Janne M Papma, Rick van Minkelen, Yolanda Pijnenburg, Myriam Barandiaran, Begoa Indakoetxea, Alazne Gabilondo, Mikel Tainta, Maria de Arriba, Ana Gorostidi, Miren Zulaica, Jorge Villanua, Zigor Diaz, Sergi Borrego-Ecija, Jaume Olives, Albert Lladó, Mircea Balasa, Anna Antonell, Nuria Bargallo, Enrico Premi, Maura Cosseddu, Stefano Gazzina, Alessandro Padovani, Roberto Gasparotti, Silvana Archetti, Sandra Black, Sara Mitchell, Ekaterina Rogaeva, Morris Freedman, Ron Keren, David Tang-Wai, Linn Öijerstedt, Christin Andersson, Vesna Jelic, Hakan Thonberg, Andrea Arighi, Chiara Fenoglio, Elio Scarpini, Giorgio Fumagalli, Thomas Cope, Carolyn Timberlake, Timothy Rittman, Christen Shoesmith, Robart Bartha, Rosa Rademakers, Carlo Wilke, Hans-Otto Karnarth, Benjamin Bender, Rose Bruffaerts, Philip Van Damme, Mathieu Vandenbulcke, Catarina B. Ferreira, Gabriel Miltenberger, Carolina Maruta, Ana Verdelho, Sónia Afonso, Ricardo Taipa, Paola Caroppo, Giuseppe Di Fede, Giorgio Giaccone, Sara Prioni, Veronica Redaelli, Giacomina Rossi, Pietro Tiraboschi, Diana Duro, Maria Rosario Almeida, Miguel Castelo-Branco, Maria João Leitão, Miguel Tabuas-Pereira, Beatriz Santiago, Serge Gauthier, Pedro Rosa-Neto, Michele Veldsman, Paul Thompson, Tobias Langheinrich, Catharina Prix, Tobias Hoegen, Elisabeth Wlasich, Sandra Loosli, Sonja Schonecker, Elisa Semler, Sarah Anderl-Straub, Luisa Benussi, Giuliano Binetti, Michela Pievani, Gemma Lombardi, Benedetta Nacmias, Camilla Ferrari, Valentina Bessi, Cristina Polito.

## Supplementary Material

fcaa122_Supplementary_Data

## Abbreviations

*C9orf72* =: chromosome 9 open reading frame 72
EWCE =: expression-weighted cell-type enrichment
FDR =: false discovery rate
FTD =: frontotemporal dementia
GENFI =: genetic FTD initiative
GFAP =: glial fibrillary acidic protein
GO =: Gene Ontology
*GRN* =: progranulin
MAPT =: microtubule-associated tau gene
OR =: odds ratio
*TREM2* =: triggering receptor expressed on myeloid cells 2
VBM =: voxel-based morphometry
