# Supplementary material for: Analysis of brain atrophy and local gene expression in genetic frontotemporal dementia
Source: Brain Commun. 2020 Aug 19;2(2):fcaa122. doi: 10.1093/braincomms/fcaa122 (PMC7667525; doi:10.1093/braincomms/fcaa122)
Supplement: fcaa122_Supplementary_Data [file fcaa122_supplementary_data.zip › Supplementary_material.docx]

**Supplementary Material**

**Analysis of brain atrophy and local gene expression in genetic frontotemporal dementia**

Andre Altmann, David M Cash, Martina Bocchetta, Carolin Heller, Regina Reynolds, Katrina Moore, Rhian S Convery, David L Thomas, John C van Swieten, Fermin Moreno, Raquel Sanchez-Valle, Barbara Borroni, Robert Laforce Jr, Mario Masellis, Maria Carmela Tartaglia, Caroline Graff, Daniela Galimberti, James B Rowe, Elizabeth Finger, Matthis Synofzik, Rik Vandenberghe, Alexandre de Mendonça, Fabrizio Tagliavini, Isabel Santana, Simon Ducharme, Chris R Butler, Alex Gerhard, Johannes Levin, Adrian Danek, Giovanni Frisoni, Roberta Ghidoni, Sandro Sorbi, Markus Otto, Mina Ryten, Jonathan D Rohrer, on behalf of the Genetic FTD Initiative, GENFI.*

**Corresponding author:**

Dr. Andre Altmann

Email: a.altmann@ucl.ac.uk

**Description of additional datasets**

**Dataset S1: *Results from the spatial association analysis.*** This table shows for every eligible probe, the mapped gene name, the z-score of the meta-analysis for the six donor brains, the corresponding P-values and the FDR adjusted P-value for associations with the three separate t-maps.

**Dataset S2: *Enrichments for pathways.*** Each sheet in this table shows the results of the enrichment analysis for the six gene lists (genes positively and negatively correlated with atrophy, respectively) and the two consensus lists. Columns represent the pathway name, the number of overlapping genes between list and pathway, the expected number of overlapping genes, the odds ratio (OR) and the overrepresentation p-value (including FDR-correction).

**Dataset S3: *Enrichments for brain six cell-types.*** Enrichment analysis testing gene lists with significant positive or negative association for cell-type marker genes. Human cell-type gene lists for neurons (N), microglia (MG), mature astrocytes (MA), endothelial cells (EC), oligodendrocytes (OLG) and oligodendrocyte precursor cells (OPC) were obtained from Zhang et al. (2016).

**Dataset S4: *Fine-grained EWCE results* of the two consensus lists.** The columns provide the cell-type abbreviation, the broad cell class (oligodendrocyte, neuron, astrocytes, ependymal, vascular and immune), a more detailed description of the cell type followed by enrichment statistics for the negative consensus and the positive consensus list, respectively. P-values were obtained using 100,000 permutation**s.**

**List of GENFI consortium authors**

Caroline Greaves BSc^1^, Georgia Peakman MSc^1^, Rachelle Shafei MRCP^1^, Emily Todd MRes^1^, Martin N. Rossor MD FRCP^1^, Jason D. Warren PhD FRACP^1^, Nick C. Fox MD FRCP^1,2^, Henrik Zetterberg^2^, Rita Guerreiro PhD^3^, Jose Bras PhD^3^, Jennifer Nicholas PhD^4^, Simon Mead PhD^5^, Lize Jiskoot PhD^6^, Lieke Meeter MD^6^, Jessica Panman MSc^6^, Janne M Papma PhD^6^, Rick van Minkelen PhD^7^, Yolanda Pijnenburg PhD^8^, Myriam Barandiaran PhD^9,10^, Begoña Indakoetxea MD^9,10^, Alazne Gabilondo MD^10^, Mikel Tainta MD^10^, Maria de Arriba BSc^10^, Ana Gorostidi PhD^10^, Miren Zulaica BSc^10^, Jorge Villanua MD PhD^11^ Zigor Diaz^12^, Sergi Borrego-Ecija MD^13^, Jaume Olives MSc^13^, Albert Lladó PhD^13^, Mircea Balasa PhD^13^, Anna Antonell PhD^13^, Nuria Bargallo PhD^14^, Enrico Premi MD^15^, Maura Cosseddu MPsych^15^, Stefano Gazzina MD^15^, Alessandro Padovani MD PhD^15^, Roberto Gasparotti MD^16^, Silvana Archetti MBiolSci^17^, Sandra Black MD^19^, Sara Mitchell MD^19^, Ekaterina Rogaeva PhD^20^, Morris Freedman MD^21^, Ron Keren MD^22^, David Tang-Wai MD^23^, Linn Öijerstedt MD^24^, Christin Andersson PhD^25^, Vesna Jelic MD^26^, Hakan Thonberg MD^27^, Andrea Arighi MD^28,29^, Chiara Fenoglio PhD^28,29^, Elio Scarpini MD^28,29^, Giorgio Fumagalli MD^28,29^, Thomas Cope MRCP^30^, Carolyn Timberlake BSc^30^, Timothy Rittman MRCP^30^, Christen Shoesmith MD^31^, Robart Bartha PhD^32,33^, Rosa Rademakers PhD^34^, Carlo Wilke MD^35,36^, Hans-Otto Karnarth MD^37^, Benjamin Bender MD^38^, Rose Bruffaerts MD PhD^39^, Philip Van Damme MD PhD^40^, Mathieu Vandenbulcke MD PhD^41,42^, Catarina B. Ferreira MSc^43^, Gabriel Miltenberger PhD^44^, Carolina Maruta MPsych PhD^45^, Ana Verdelho MD PhD^46^, Sónia Afonso BSc^47^, Ricardo Taipa MD PhD^48^, Paola Caroppo MD PhD^49^, Giuseppe Di Fede MD PhD^49^, Giorgio Giaccone MD^49^, Sara Prioni PsyD^49^, Veronica Redaelli MD^49^, Giacomina Rossi MSc^49^, Pietro Tiraboschi MD^49^, Diana Duro NPsych^50^, Maria Rosario Almeida PhD^50^, Miguel Castelo-Branco MD PhD^50^, Maria João Leitão BSc^51^, Miguel Tabuas-Pereira MD^52^, Beatriz Santiago MD^52^, Serge Gauthier MD^53^, Pedro Rosa-Neto MD PhD^54^**,** Michele Veldsman PhD^55^, Paul Thompson^56^, Tobias Langheinrich^56^, Catharina Prix MD^57^, Tobias Hoegen MD^57^, Elisabeth Wlasich Mag. rer. nat.^57^, Sandra Loosli MD^57^, Sonja Schonecker MD^57^, Elisa Semler Dr.hum.biol Dipl. Psych^58^, Sarah Anderl-Straub Dr.hum.biol Dipl.Psych^58^, Luisa Benussi PhD^59^, Giuliano Binetti MD^59^, Michela Pievani PhD^59^, Gemma Lombardi MD^60^, Benedetta Nacmias PhD^60^, Camilla Ferrari^60^, Valentina Bessi^60^, Cristina Polito^61^.

**Affiliations**

^1^Dementia Research Centre, Department of Neurodegenerative Disease, UCL Queen Square Institute of Neurology, London, UK; ^2^Dementia Research Institute, Department of Neurodegenerative Disease, UCL Institute of Neurology, Queen Square, London, UK; ^3^Center for Neurodegenerative Science, Van Andel Research Institute, Grand Rapids, Michigan, USA.^4^Department of Medical Statistics, London School of Hygiene and Tropical Medicine, London, UK; ^5^MRC Prion Unit, Department of Neurodegenerative Disease, UCL Institute of Neurology, Queen Square, London, UK; ^6^Department of Neurology, Erasmus Medical Centre, Rotterdam, Netherlands; ^7^Department of Clinical Genetics, Erasmus Medical Centre, Rotterdam, Netherlands; ^8^Amsterdam University Medical Centre, Amsterdam VUmc, Amsterdam, Netherlands; ^9^Cognitive Disorders Unit, Department of Neurology, Donostia University Hospital, San Sebastian, Gipuzkoa, Spain; ^10^Neuroscience Area, Biodonostia Health Research Institute, San Sebastian, Gipuzkoa, Spain; ^11^OSATEK, University of Donostia, San Sebastian, Gipuzkoa, Spain; ^12^CITA Alzheimer, San Sebastian, Gipuzkoa, Spain; ^13^Alzheimer’s disease and Other Cognitive Disorders Unit, Neurology Service, Hospital Clínic, Barcelona, Spain; ^14^Imaging Diagnostic Center, Hospital Clínic, Barcelona, Spain; ^15^Centre for Neurodegenerative Disorders, Neurology Unit, Department of Clinical and Experimental Sciences, University of Brescia, Brescia, Italy; ^16^Neuroradiology Unit, University of Brescia, Brescia, Italy; ^17^Biotechnology Laboratory, Department of Diagnostics, Spedali Civili Hospital, Brescia, Italy; ^18^Clinique Interdisciplinaire de Mémoire Département des Sciences Neurologiques Université Laval Québec, Quebec, Canada; ^19^Sunnybrook Health Sciences Centre, Sunnybrook Research Institute, University of Toronto, Toronto, Canada; ^20^Tanz Centre for Research in Neurodegenerative Diseases, University of Toronto, Toronto, Canada; ^21^Baycrest Health Sciences, Rotman Research Institute, University of Toronto, Toronto, Canada; ^22^The University Health Network, Toronto Rehabilitation Institute, Toronto, Canada; ^23^The University Health Network, Krembil Research Institute, Toronto, Canada; ^24^Center for Alzheimer Research, Division of Neurogeriatrics, Department of Neurobiology, Care Sciences and Society, Bioclinicum, Karolinska Institutet, Solna, Sweden; Unit for Hereditary Dementias, Theme Aging, Karolinska University Hospital, Solna, Sweden; ^25^Department of Clinical Neuroscience, Karolinska Institutet, Stockholm, Sweden; ^26^Division of Clinical Geriatrics, Karolinska Institutet, Stockholm, Sweden; ^27^Center for Alzheimer Research, Divison of Neurogeriatrics, Karolinska Institutet, Stockholm, Sweden; ^28^Fondazione IRCCS Ca’ Granda Ospedale Maggiore Policlinico, Neurodegenerative Diseases Unit, Milan, Italy; ^29^University of Milan, Centro Dino Ferrari, Milan, Italy; ^30^Department of Clinical Neurosciences, University of Cambridge, Cambridge, UK; ^31^Department of Clinical Neurological Sciences, University of Western Ontario, London, Ontario Canada; ^32^Department of Medical Biophysics, The University of Western Ontario, London, Ontario, Canada; ^33^Centre for Functional and Metabolic Mapping, Robarts Research Institute, The University of Western Ontario, London, Ontario, Canada; ^34^Department of Neuroscience, Mayo Clinic, Jacksonville, Florida, USA; ^35^Department of Neurodegenerative Diseases, Hertie-Institute for Clinical Brain Research and Center of Neurology, University of Tübingen, Tübingen, Germany; ^36^Center for Neurodegenerative Diseases (DZNE), Tübingen, Germany; ^37^Division of Neuropsychology, Hertie-Institute for Clinical Brain Research and Center of Neurology, University of Tübingen, Tübingen, Germany; ^38^Department of Diagnostic and Interventional Neuroradiology, University of Tübingen, Tübingen, Germany; ^39^Laboratory for Cognitive Neurology, Department of Neurosciences, KU Leuven, Leuven, Belgium; ^40^Neurology Service, University Hospitals Leuven, Belgium, Laboratory for Neurobiology, VIB-KU Leuven Centre for Brain Research, Leuven, Belgium; ^41^Geriatric Psychiatry Service, University Hospitals Leuven, Belgium; ^42^Neuropsychiatry, Department of Neurosciences, KU Leuven, Leuven, Belgium; ^43^Laboratory of Neurosciences, Institute of Molecular Medicine, Faculty of Medicine, University of Lisbon, Lisbon, Portugal; ^44^Faculty of Medicine, University of Lisbon, Lisbon, Portugal; ^45^Laboratory of Language Research, Centro de Estudos Egas Moniz, Faculty of Medicine, University of Lisbon, Lisbon, Portugal; ^46^Department of Neurosciences and Mental Health, Centro Hospitalar Lisboa Norte - Hospital de Santa Maria & Faculty of Medicine, University of Lisbon, Lisbon, Portugal; ^47^Instituto Ciencias Nucleares Aplicadas a Saude, Universidade de Coimbra, Coimbra, Portugal; ^48^Neuropathology Unit and Department of Neurology, Centro Hospitalar do Porto - Hospital de Santo António, Oporto, Portugal; ^49^Fondazione IRCCS Istituto Neurologico Carlo Besta, Milano, Italy; ^50^Faculty of Medicine, University of Coimbra, Coimbra, Portugal; ^51^Centre of Neurosciences and Cell biology, Universidade de Coimbra, Coimbra, Portugal; ^52^Neurology Department, Centro Hospitalar e Universitario de Coimbra, Coimbra, Portugal; ^53^Alzheimer Disease Research Unit, McGill Centre for Studies in Aging, Department of Neurology & Neurosurgery, McGill University, Montreal, Québec, Canada; ^54^Translational Neuroimaging Laboratory, McGill Centre for Studies in Aging, McGill University, Montreal, Québec, Canada; ^55^Nuffield Department of Clinical Neurosciences, Medical Sciences Division, University of Oxford, Oxford, UK; ^56^Division of Neuroscience and Experimental Psychology, Wolfson Molecular Imaging Centre, University of Manchester, Manchester, UK; ^57^Neurologische Klinik, Ludwig-Maximilians-Universität München, Munich, Germany; ^58^Department of Neurology, University of Ulm, Ulm; ^59^Instituto di Ricovero e Cura a Carattere Scientifico Istituto Centro San Giovanni di Dio Fatebenefratelli, Brescia, Italy; ^60^Department of Neuroscience, Psychology, Drug Research, and Child Health, University of Florence, Florence, Italy. ^61^Department of Biomedical, Experimental and Clinical Sciences “Mario Serio”, Nuclear Medicine Unit, University of Florence, Florence, Italy.
